# Atomic dynamics of stress-induced lattice misalignment structures in a KDP subsurface

**DOI:** 10.1039/d0ra01291b

**Published:** 2020-06-23

**Authors:** Y. Hu, Z. Zhu, H. Z. Shao, J. M. Xiao, M. Xu, L. Zhao, J. Zhuang

**Affiliations:** Shanghai Engineering Research Center of Ultra-Precision Optical Manufacturing, Department of Optical Science and Engineering, Fudan University Shanghai 200433 China junzhuang@fudan.edu.cn; State Key Laboratory of Surface Physics, Department of Physics, Fudan University Shanghai 200433 China lizhao@fudan.edu.cn; College of Electrical and Electronic Engineering, Wenzhou University Wenzhou 325035 China

## Abstract

We present an *ab initio* molecular dynamics study of the thermal stability and dynamics behaviors of lattice misalignment structures (LMSs) in the subsurface layers of KH_2_PO_4_ (KDP) crystals. The dehydration process at the atomic scale is observed in the LMS system, which is the same as that in a perfect KDP crystal. However, the paths entering the dehydration process are various. The interesting result is that compared with a perfect KDP crystal, many new paths appear in the LMS system, and even in the same paths, the dehydration is more likely to happen in the LMS system. This leads to a dramatic increase in the dehydration numbers in the LMS system, for which the reasons are given in terms of structural deformation and/or uneven distribution of protons. The results elucidate the underlying atomic mechanism of the effect of LMS defects on the thermal stability of KDP material.

## Abstract 2

Compared with perfect KDP (PS), the dehydration paths and dehydration numbers in the lattice misalignment structures (LMS_1_, LMS_2_) increase significantly.

## Graphical abstract

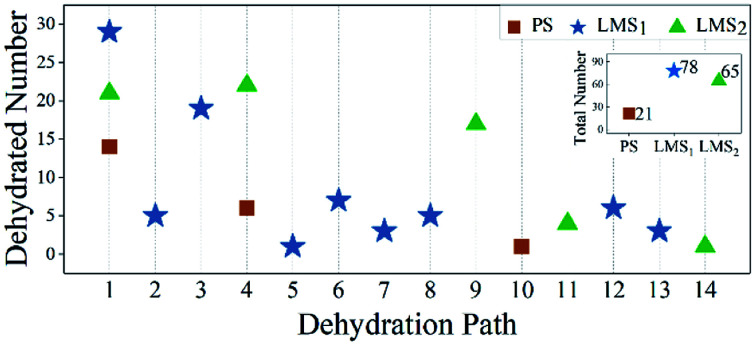

## Introduction

Potassium dihydrogen phosphate (KDP, KH_2_PO_4_) crystals have attracted intensive interest in the field of optical materials, due to their remarkable nonlinear electro-optical properties^1^ and capability of growing to large size,^2–5^ leading to many applications in various high-power large-aperture laser systems such as inertial confinement fusion reactors.^6,7^ However, the actual laser damage threshold (LDT) of a KDP crystal is usually far below its intrinsic damage threshold, which limits the output power of laser systems.^8–13^ It is widely accepted that various defects, including those in the surface/subsurface layers created during KDP lens surfacing, are the main reason for the decline of LDT of KDP.^11–18^ These defects may have an impact on the distribution of light and thus affect the LDT of KDP.^19–24^ Therefore, besides growing KDP crystals close to perfection, it is also important to obtain smoother surfaces and more damage-free subsurfaces after machining to increase the LDT of KDP.

In recent years, with the improvement of machining technique, a smoother surface of KDP can be produced by different ultra-precision surface machining methods, such as single point diamond turning,^25–27^ ultra-precision grinding^28,29^ and magnetorheological finishing.^30^ Even so, after machining, the damage at a more microscopic level still cannot be avoided as expected. Recently, researchers evaluated more precisely the subsurface structure of a KDP crystal after ultra-precision machining by utilizing grazing incidence X-ray diffraction technique. The results show that a shallow subsurface layer of a machined KDP crystal has turned to a lattice misalignment structure (LMS).^31,32^ This means that, compared with other machining-induced defects,^11,28^ such as pits, cracks, and nicks, such stress-induced subsurface micro “LMS” defect seems impossible to avoid, even with a very advanced surface machining technique. Therefore, clarifying the characteristics of LMS, a kind of “intrinsic” defect or damage, and its effect on the LDT of KDP becomes more important. We have previously published an *ab initio* study of the lattice misalignment structures in KDP crystals. In this previous work, by utilizing the *ab initio* static calculations, we found out for the first time various stable lattice misalignment structures by applying stress to KDP crystals, and calculated their electronic band structures and optical absorption properties. The results show that the electronic band structure and the optical absorption of misalignment structure indeed have some differences compared to a perfect KDP crystal.^33^

Compared with the static study, the dynamic study on the evolution of the misalignment structure, however, is more important, since it directly determines the material thermal stability under the action of light and heat. This is particularly significant in large-aperture laser systems, where micro-defects may not hinder the device performance, but the growth and even decomposition of defects are catastrophic.^34–37^ In the present work, we picked two stable LMSs from our previous static calculations and studied the atomic dynamics behaviors in these misalignment structures by utilizing *ab initio* molecular dynamics (MD) simulations. We want to see the atomic-level dynamic evolution of LMS, the possible structural damage, the damage pathway and how difficult it is compared with the perfect structure of KDP, and so on. These results are helpful to evaluate the possible adverse effects of such stress-induced “intrinsic” misalignment structure during the working process of KDP components.

## Model and method

**Fig. 1:**
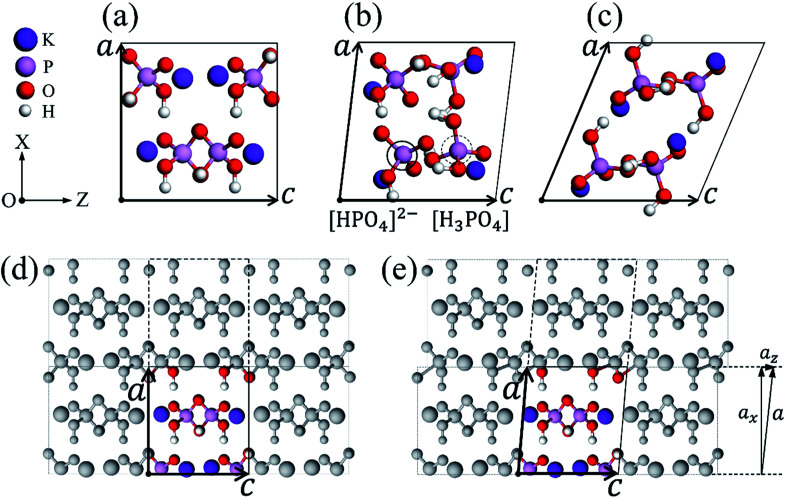
The unit cell of a perfect KDP crystal (a). Two stable LMSs with the z-component of vector ***a*** of 8.40 Å (b) and 26.0 Å (c). The radicals labeled by the solid and dashed circles in (b) denote radicals of [HPO_4_]^2−^ and [H_3_PO_4_], respectively. The perfect KDP bulk system (d), by adjusting the z-component of vector ***a***, the grey atoms in the dashed box are moved along the *Z* direction a small displacement ***a***_z_ (e). The ***a*** and ***c*** indicate the basis vectors of the unit cell. The white, red, pink and purple balls denote H, O, P and K atoms, respectively, which are all the same in the following figures.

As shown in Fig. 1(a), we give the unit cell of a perfect KDP crystal, which consists of four KH_2_PO_4_ molecules and contains two molecule layers. The perfect cell can also be seen in Fig. 1(d), *i.e.*, the colored part, which differs from Fig. 1(a) only in the selection of unit cells; the grey parts are obtained by utilizing the periodic boundary conditions, a perfect bulk is thus formed. In our previous work,^33^ we presented a simple and effective method to obtain the atomic configuration of misalignment structure on the (200) plane. Specifically, we moved the grey atoms in the dashed box along the *Z* direction a small displacement ***a***_z_ by changing the z-component of vector ***a***, as shown in Fig. 1(d) and (e), the P–O bonds at the (200) boundary of the unit cell were thus slightly deformed [see Fig. 1(e)]. By doing so, we try to model the local strain induced by the stress resulting from the cutting tip. Then, the system was fully relaxed. Through relaxation, the stress in the deformed P–O bonds can be transferred to every layer in the system, during which the (200) LMS appears naturally. More details can be found from ref. 33. Following the similar procedures as the above, we simulated the LMS evolution and obtained lots of local stable LMSs, such as the two representative ones in Fig. 1(b) and (c). Their z-components of vector ***a*** change by 8.40 Å and 26.0 Å relative to the perfect KDP structure, as shown in Fig. 1(a)–(c). Different z-components of vector ***a*** mean different degrees of misalignment. Compared with the perfect structure (PS) of KDP, both of two LMSs produce obvious deformation [see Fig. 1(a)–(c)]. Specially, the LMS at 8.40 Å (LMS_1_) has new radicals of “[HPO_4_]^2−^” and “[H_3_PO_4_]” [see Fig. 1(b)], which is different from that in the PS and the LMS at 26.0 Å (LMS_2_).

In the present work, aiming at the two LMSs in Fig. 1(b) and (c), we use *ab initio* MD simulations to study their dynamics behaviors. For comparison, the perfect KDP structure is also studied under the same conditions. We first generate the 2 × 2 × 2 supercells of PS and two LMSs. Then, the three supercells including 256 atoms are fully relaxed. After that, the three optimized systems are heated at a constant temperature of 2000 K. It should be noted that an unreasonable high temperature adopted here for simulation is just to accelerate the chemical reaction, since the simulated time scale (ps) is too short for the chemical processes. The similar practice can be widely found in the literatures.^38–42^ A step size of 1.0 fs is used for all MD calculations with each simulation running for approximately 5000 steps.^43^ In all cases, dynamical equilibrium (based on the total system energy) appears to be reached in about 500 steps. After the systems reach to the dynamical equilibrium, we begin to trace the atomic trajectories. We focus on those steps with obvious atomic position and bond changes to find out the possible structural changes/damages. Then, we further extract the obvious changed structures and fully relax them to judge whether the changed structures can be stable. To compare the difficulty of the possible structural changes/damages in the LMS and PS systems, we performed 30 independent MD simulations for each system.

All calculations including the structural relaxations and MD simulations are performed based on the density function theory (DFT) utilizing the Vienna *ab initio* simulation program pack (VASP) package.^44^ The exchange–correlation potential is described by the generalized gradient approximation (GGA) with the Perdew–Burke–Ernzerhof functional.^45^ PAW potentials are used for electron–ion interactions. The kinetic energy cutoff for the plane-wave basis is set to be 680 eV, which leads to the converged result for the total system energy better than 1 meV per atom.^21,46^ A 2 × 2 × 2 supercell with a 3 × 3 × 3 Monkhorst–Pack *k*-point mesh is used for all calculations. The conjugate gradient algorithm is used to relax the ions and the residual force on each atom is taken as the convergence criterion which is smaller than 0.01 eV Å^−1^. With these parameters, the obtained lattice parameters of the relaxed perfect KDP crystal are *a* = *b* = 7.44 Å and *c* = 6.96 Å, which are in excellent agreement with the experimental values of *a* = *b* = 7.45 Å and *c* = 6.97 Å.^47^*Ab initio* molecular dynamics (MD) calculations are achieved by the Verlet algorithm and the canonical ensemble (NVT) is adopted using the Nosé thermostat to control temperature.

## Results and discussion

**Fig. 2:**
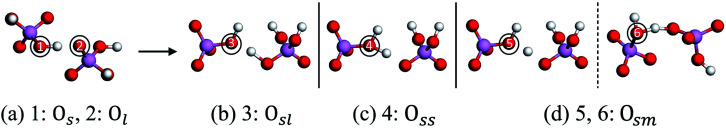
Schematic of atomic configuration of two [H_2_PO_4_]^−^ radicals in the initial PS system (a). When the systems reach to the dynamical equilibrium, various configurations (b), (c) and (d) appear in any of the three systems. The oxygen atoms marked with black circles and Arabic numerals (1–6) denote five types of oxygen states: O_s_ and O_l_ (a), O_sl_ (b), O_ss_ (c), and O_sm_ (d). The subscripts “s” (short), “l” (long) and “m” (medium) describe the distance of proton relative to two oxygen atoms in the O–H⋯O or O⋯H⋯O (O–H–O) bonds, respectively.

Based on the obtained MD trajectories, we first focus on the atomic dynamic evolution in the PS and LMS systems. It is known that in the static perfect KDP structure (paraelectric phase), each oxygen atom connects only one close (O–H) or far (O⋯H) proton through the H-bond (O–H⋯O).^48–53^ We will refer to the oxygen atom that connects the proton through the short distance as “O_s_” (O–H), and the other case as “O_l_” (O⋯H), as shown in Fig. 2(a). In sharp contrast, in the dynamic evolution, as shown in Fig. 2(b)–(d), the oxygen atoms that connect two protons appear in the three systems because of the rotation and movement of PO_4_ tetrahedrons, the transfer of protons, and the change in the H-bond length. Here, we use the similar symbols like “O_sl_, O_ss_ and O_sm_” to describe these new oxygen states; the subscript “m” (medium) indicates that the proton is approximately located at the midpoint position in the H-bond (O⋯H⋯O/O–H–O), as shown in Fig. 2(d). Another obvious dynamic phenomenon observed in the three systems, especially for the PS and LMS_2_ systems, is that the proton may escape from one PO_4_ tetrahedron to another, turning two [H_2_PO_4_]^−^ radicals into one [HPO_4_]^2−^ radical and one [H_3_PO_4_] radical. Lastly but most importantly, when the oxygen state O_sl_ appears, the special dynamics behavior that probably results in a qualitative change in material usually occurs in the subsequent dynamic evolution. That is dehydration reaction by which some radicals will be decomposed.

**Fig. 3:**
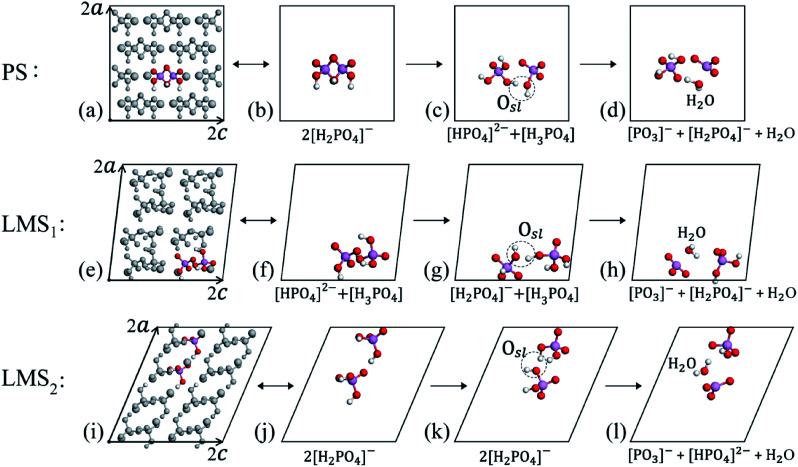
The supercells of PS (a), LMS_1_ (e), and LMS_2_ (i), where the colored atoms are referred to as the “initial combination”. For clarity, we only display the initial combinations in (b), (f), and (j). After a series of dynamic evolution, the initial combinations produce obvious changes in structure and/or chemical composition, and generate the oxygen state “O_sl_”; we will refer to such changed initial combinations as the “dehydrated combinations” (c), (g), and (k). In the subsequent evolution, the oxygen state “O_sl_” evolves into a water molecule (d), (h), and (l). The ***a*** and ***c*** denote the basis vectors of the unit cell.

We find that the oxygen state O_sl_ can appear in various possible combinations of PO_4_ tetrahedrons and protons, and they all have the potential to dehydrate. In Fig. 3, we give the three instances that correspond to three dehydration paths. Fig. 3(a) displays the PS system. Here, we take the colored atoms (2[H_2_PO_4_]^−^) as an example, which will be referred to as the “initial combination”. For clarity, we remove the grey atoms in the PS system and only display the initial combination [see Fig. 3(b)]. Through a series of dynamic evolution, as shown in Fig. 3(b) and (c), the initial combination of “2[H_2_PO_4_]^−^” turns into the combination of “[HPO_4_]^2−^ + [H_3_PO_4_]”, owing to the transfer of protons; besides, the rotation and movement of PO_4_ tetrahedrons lead to the formation of the oxygen state “O_sl_” [see Fig. 3(c)]. The formation of oxygen state “O_sl_” is an important dynamic evolution result, since “O_sl_” may evolve into a water molecule in the subsequent evolution, which results in a qualitative change in KDP material [see Fig. 3(d)]. We will refer to the combination in Fig. 3(c) that is going to dehydrate as the “dehydrated combination”. Another two examples for the LMS_1_ and LMS_2_ systems are given in Fig. 3(e)–(h) and (i)–(l), respectively. Similarly, after a series of dynamic evolution, besides the obvious changes in structure and/or chemical composition, the oxygen state “O_sl_” appears in the dehydrated combinations [see Fig. 3(g) and (k)]. Significantly, the oxygen state “O_sl_” could also evolve into a water molecule in the subsequent evolution [see Fig. 3(h) and (l)], and the dynamics process from “O_sl_” to “H_2_O” are fully identical to that in the PS system.

Certainly, in the MD simulations, there are more than three types of dehydration paths. In Table 1, we give all the dehydration paths observed in the three systems of PS, LMS_1_, and LMS_2_, which are numbered by the Arabic numeral “1–14”. The important result, as seen in the second column in Table 1, only three dehydration paths “1, 4, and 10” are in the PS system; the others are all in the LMS systems. In Table 1, we also give the corresponding initial combinations, which are labelled by the symbols of “A_1_, A_2_, A_3_, B_1_, …” (see the third column). In the subsequent dynamic evolution, the initial combinations evolve into the dehydrated combinations, in which “O_sl_” is formed. As seen in Table 1, some initial combinations evolve into the same dehydrated combination. For example, the initial combinations “A_1_, A_2_, and A_3_” all become into the dehydrated combination “A”. Therefore, there are only six types of dehydrated combinations, which are labeled by the capital letters from “A” to “F” in Table 1. Interestingly, after the formation of “O_sl_”, it is through the same dynamics process that the oxygen states “O_sl_” in the dehydrated combinations evolve into the water molecules. That is, from the six types of dehydrated combinations to the final dehydrated products, see the fourth to sixth columns in Table 1, the dynamics process from “O_sl_” to the “water molecule” are completely the same, indicated by s_1_–s_5_.

**Table 1:** The relevant information of the fourteen dehydration paths in the three systems. The initial combinations at s_0_ step labeled by the symbols of “A_1_, A_2_, A_3_, B_1_, …” are given in the third column. The dehydrated combinations at s_1_ step are labeled by the capital letters from A to F in the fourth column. In the subsequent evolution, the oxygen states “O_sl_” in the dehydrated combinations all evolve into the water molecules through the same dynamics process, namely “s_1_–s_5_”. The dehydrated products are given in the last column

Path	System	Initial combination (s_0_)	Dehydrated combination (s_1_)	Process	Products (s_4_, s_5_)
1	PS, LMS_1_, LMS_2_	A_1_: 2[H_2_PO_4_]^−^	A: [HPO_4_]^2−^ + [H_3_PO_4_]	s_1_–s_5_	H_2_O, [PO_3_]^−^, [P_2_O_7_]^4−^
2	LMS_1_	A_2_: [HPO_4_]^2−^ + [H_2_PO_4_]^−^			
3	LMS_1_	A_3_: [H_2_PO_4_]^−^ + [H_3_PO_4_]			
4	PS, LMS_2_	B_1_: 2[H_2_PO_4_]^−^	B: 2[H_2_PO_4_]^−^		
5	LMS_1_	B_2_: 2[H_3_PO_4_]			
6	LMS_1_	C_1_: [HPO_4_]^2−^ + [H_3_PO_4_]	C: [H_2_PO_4_]^−^ + [H_3_PO_4_]		
7	LMS_1_	C_2_: [HPO_4_]^2−^ + [H_2_PO_4_]^−^			
8	LMS_1_	C_3_: [H_2_PO_4_]^−^ + [H_3_PO_4_]			
9	LMS_2_	C_4_: 2[H_2_PO_4_]^−^			
10	PS	D_1_: 2[H_2_PO_4_]^−^	D: 2[HPO_4_]^2−^		
11	LMS_2_	E_1_: 2[H_2_PO_4_]^−^	E: [HPO_4_]^2−^ + [H_2_PO_4_]^−^		
12	LMS_1_	E_2_: [H_2_PO_4_]^−^ + [H_3_PO_4_]			
13	LMS_1_	E_3_: [HPO_4_]^2−^ + [H_2_PO_4_]^−^			
14	LMS_2_	F_1_: 3[H_2_PO_4_]^−^	F: [HPO_4_]^2−^ + [H_2_P_2_O_7_]^2−^		

**Fig. 4:**
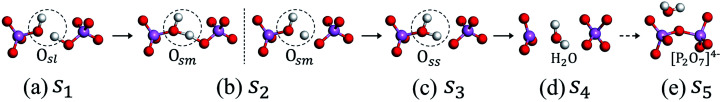
The atomic dynamic evolution from the dehydrated combination (s_1_) to the final products (s_4_, s_5_), where only the two protons that form water are given. In the s_1_ step, O_sl_ appears in the dehydrated combination (a); then in the s_2_ (b) and s_3_ (c) steps, O_sl_ evolves into O_sm_ and then O_ss_; the oxygen states are marked by the grey dashed circles; finally, in the s_4_ step (d), “O_ss_” liberates from the PO_4_ tetrahedron in the form of a water molecule and leaving behind a [PO_3_]^−^ radical. In the s_5_ step, the [PO_3_]^−^ radical interacts with an adjacent [PO_4_]^3−^ radical to form a [P_2_O_7_]^4−^ radical (e).

In Fig. 4, we display the atomic dynamic evolution in the dehydration process, step s_1_ to s_5_, where only the two protons that form water are given for clarity. In the “s_1_” step, the oxygen state O_sl_ appears in the “dehydrated combination”. Then, in the s_2_ step, the H-bond gets shorter/longer, turning the O⋯H–O bond into the O–H–O/O⋯H⋯O bond; this results in the formation of oxygen state O_sm_. Next, in the s_3_ step, the O–H–O/O⋯H⋯O bond evolves into the O–H⋯O bond, during which the proton successfully escapes from one oxygen atom to the other, the oxygen state O_ss_ thus formed. Finally, the most remarkable change occurs in the s_4_ step, that is, the newly formed oxygen state O_ss_ is ejected from the PO_4_ tetrahedron as a water molecule and leaving behind a [PO_3_]^−^ radical. By the way, the newly formed [PO_3_]^−^ radical may interact with a nearest [PO_4_]^3−^ radical to form a [P_2_O_7_]^4−^ radical in the subsequent dynamic evolution [see Fig. 4(e)]. Indeed, researchers have detected the intermediate product [P_2_O_7_]^4−^ radical in the direct heating of KDP crystals above 500 K.^54,55^ Furthermore, we extract the structures in the s_4_ and s_5_ steps from the MD trajectories and fully relax them, to judge whether the water molecules can exist stably; the results show that the water molecules in both two steps still exist stably after relaxation. In summary, despite the paths (1–14) entering the dehydration process are various, their dehydration processes from “O_sl_” to the “water molecule” are the same; that is, after the formation of “O_sl_”, it first evolves into “O_sm_”, then turns into “O_ss_”, eventually the oxygen state “O_ss_” liberates from the PO_4_ tetrahedron in the form of a water molecule. If only the two protons that form water are considered, the dehydration process can be described as the following equations, which are consistent with the experimental observations.^54,55^1
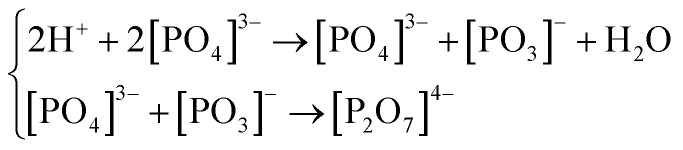


**Fig. 5:**
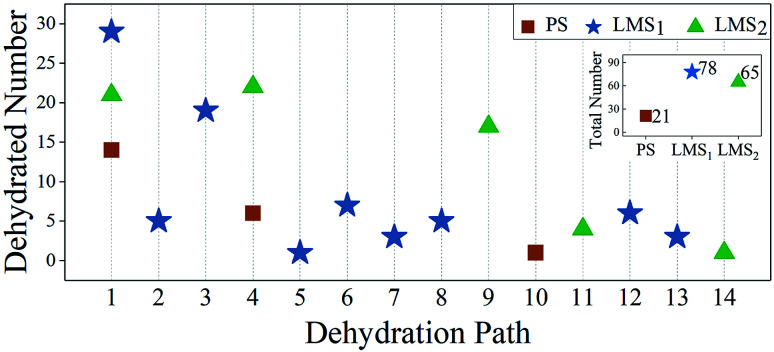
Statistical results for the dehydrated numbers in the PS (red rectangles), LMS_1_ (blue stars), and LMS_2_ (green triangles) systems. The abscissas (1–14) denote fourteen dehydration paths observed in the three systems (see Table 1). Inset: the total dehydrated numbers for the three systems.

To compare the decomposition difficulty of LMS and PS systems, as shown in Fig. 5, we give the statistical results for the dehydrated numbers of each system in the 30 MD simulations. From Fig. 5, we can see that, compared with the PS system, the dehydration paths in the LMS systems increase significantly, especially in the LMS_1_ system. Specifically, only three dehydration paths are in the PS system (paths 1, 4, and 10); in sharp contrast, there are nine and five dehydration paths in the LMS_1_ and LMS_2_ systems, respectively (see Fig. 5). For the LMS_1_ system, this result should be mainly attributed to its initial chemical composition. Indeed, in the three initial systems, only the LMS_1_ system has different radicals: [HPO_4_]^2−^, [H_2_PO_4_]^−^, and [H_3_PO_4_]. These different radicals result in the formation of some unique dehydration paths. For example, in the LMS_1_ system, there are some dehydration paths that start from the initial combinations which consist of two different radicals, such as “[HPO_4_]^2−^ + [H_2_PO_4_]^−^”, “[H_2_PO_4_]^−^ + [H_3_PO_4_]”, *etc.* (see Table 1); while in the PS and LMS_2_ systems, there are no such dehydration paths. For the LMS_2_ system, the increased dehydration paths should be caused by the deformed structure. As shown in Fig. 1(a)–(c), the H-bonds in the LMSs are obviously stretched compared with that in the PS. The stretched H-bonds decrease the attraction between the adjacent PO_4_ tetrahedrons, making it easier for the PO_4_ tetrahedrons to move and rotate. This catalyzes the scission of the H-bond network, making the consequence that the protons in the LMS systems have more transfer degrees of freedom, thus generating more types of dehydration paths in the LMS_2_ system.

Another interesting result in Fig. 5 is that, in the same dehydration paths, such as path 1 and path 4, the dehydrated numbers for the LMS systems are obviously greater than that of the PS system. There are two possible reasons: on one hand, the PO_4_ tetrahedrons in the LMS systems are easier to move and rotate due to the stretched H-bonds, so that the LMS systems are easier to generate the oxygen state “O_sl_” compared with the PS system. On the other hand, the protons are unevenly distributed in the initial LMS_1_ system. The uneven distribution of protons results in the formation of some radicals in local area with charged states. The radical in the positively state, such as [KH_3_PO_4_]^+^, is apt to emit a proton outward, while the radical in the negatively state, such as [KHPO_4_]^−^, tends to contribute one [OH]^−^. That is, the uneven distribution of protons catalyzes the occurrence of dehydration.

The above facts show that, compared with the PS system, not only do many new dehydration paths appear in the LMS systems, but also the dehydrated numbers of LMS systems increase significantly in the same dehydration paths (see Fig. 5). Therefore, the total dehydrated numbers for both LMS systems are much greater than that of the PS system. As shown in the inset in Fig. 5, for the LMS_1_ and LMS_2_ systems, there are 78 and 65 dehydrations in the 30 MD simulations with each running for 5000 steps; this is in sharp contrast with the 21 dehydrations in the PS system.

**Fig. 6:**
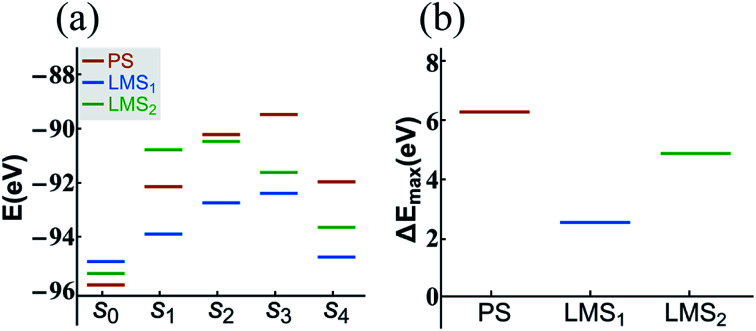
The energy level comparison diagram (a) of dehydration path 1 in the PS (red lines), LMS_1_ (blue lines), and LMS_2_ (green lines) systems, where the abscissas “s_0_” and “s_1_” correspond to the initial combination “2[H_2_PO_4_]^−^” and the dehydrated combination “[HPO_4_]^2−^ + [H_3_PO_4_]”, respectively (see Table 1); “s_2_–s_4_” correspond to the corresponding configurations in dehydration path 1. For clarity, the energy difference comparison diagram (Δ*E*_max_ = *E*_max_ − *E*_s_0__) is shown in (b).

The statistical results in Fig. 5 show that the dehydration paths in the three systems are various; but interestingly, they all contain path “1”, also the numbers of radicals dehydrated through this path account for the largest proportion (see Fig. 5). Thus, we take the path “1” as an example; we calculate the formation energy of the corresponding configurations in dehydration path 1, step s_0_ to s_4_, in which all the atoms are fully relaxed. The aim is to compare the decomposition difficulty of the same dehydration path in the three systems, and thus to further understand the statistical results. In Fig. 6(a), we display the energy level comparison diagram for the PS (red lines), LMS_1_ (blue lines), and LMS_2_ (green lines) systems; the abscissas “s_0_” and “s_1_” correspond to the initial combination “A_1_” and the dehydrated combination “A”, respectively (see Table 1); “s_2_–s_4_” correspond to the corresponding configurations in dehydration path 1; the ordinate denotes the formation energy. The formation energy is calculated by the following equation:^56–58^

2 *E*_f_ = *E*_*n*_ − *E*_*n*−*x*_

where *E*_*n*_ is the total energy of the supercell; *E*_*n*−*x*_ is the total energy of the supercell except for the combinations associated with dehydration; *n* is the number of atoms in the supercell; *x* is the number of atoms associated with dehydration. From Fig. 6(a), we can see that the energy level diagrams for the three systems are obviously different. Firstly, for the LMS_1_ system, the energy difference between the first two steps (s_0_–s_1_) is lower than that for the other two systems [see Fig. 6(a)]. This means that, compared with the PS and LMS_2_ systems, the initial combination “2[H_2_PO_4_]^−^” in the LMS_1_ system is easier to turn into the dehydrated combination “[HPO_4_]^2−^ + [H_3_PO_4_]”. This should be caused by the different radicals in the initial LMS_1_ system. That is, the uneven distribution of protons in the background promotes the transformation from the “2[H_2_PO_4_]^−^” radicals to the “[HPO_4_]^2−^ + [H_3_PO_4_]” radicals. Furthermore, the most remarkable result in Fig. 6(a) is that the maximum energy difference for both LMS systems is lower than that for the PS system. For clarity, we give the energy difference comparison diagram in Fig. 6(b), where Δ*E*_max_ = *E*_max_ − *E*_s_0__, and *E*_max_ corresponds to the maximum formation energy at s_2_ or s_3_ step. From Fig. 6(b), we can clearly see that the energy barrier for the initial combination “2[H_2_PO_4_]^−^” in the LMS_1_ system to dehydrate is lowest, which is consistent with the statistical results in Fig. 5.

In summary, compared with the PS system, the LMS systems have more dehydration paths, and even in the same dehydration paths, the LMS systems have lower energy barrier to dehydrate. The results reveal that the LMS defect decomposes more easily than the perfect KDP structure, suggesting that the thermal stability of KDP material would be much affected by such stress-induced subsurface micro LMS defect. Indeed, it has been discovered that the laser-induced or thermal-induced decomposition reactions in general begin with the local reactions in the surface/subsurface layers of KDP.^37,54,55,59^ We believe that the LMS defect may play an important role in the decomposition of the surface/subsurface layers of KDP components, and also may be one of the possible reasons for the formation of the laser-induced damages in the use of KDP.

## Conclusion

The LMS, which appears in the machining process of KDP, is investigated regarding the dynamics behaviors by utilizing the *ab initio* MD simulations. The details of atomic dynamic evolution in the three systems of PS, LMS_1_ and LMS_2_ are obtained. Especially, the dehydration phenomenon is observed, and its process is the same in the three systems. The dehydration process includes the formation of “O_sl_”, its transformation, and ejection in the form of a water molecule. However, the paths entering the dehydration process are various. Fourteen dehydration paths are observed in the three systems. The interesting result is that compared with the PS system, a lot of new dehydration paths appear in the LMS systems, and even in the same dehydration paths, the dehydration is more likely to occur in the LMS systems. This leads to the consequence that the total dehydrated numbers in the LMS systems are much greater than that in the PS system, which can be attributed to the deformed structure and/or the uneven distribution of protons. These results suggest that once the LMS defect resulting from processing appears in KDP crystals, the possibility of material decomposition will be greatly increased, which will much affect the damage threshold of KDP components.

## Conflicts of interest

There are no conflicts to declare.

## Supplementary Material
